# Single-step process to improve the mechanical properties of carbon nanotube yarn

**DOI:** 10.3762/bjnano.9.52

**Published:** 2018-02-13

**Authors:** Maria Cecilia Evora, Xinyi Lu, Nitilaksha Hiremath, Nam-Goo Kang, Kunlun Hong, Roberto Uribe, Gajanan Bhat, Jimmy Mays

**Affiliations:** 1 Institute for Advanced Studies- IEAV/DCTA, São Jose dos Campos, SP 12228, Brazil; 2 Department of Chemistry- University of Tennessee, Knoxville, TN 37996, USA; 3 Department of Materials Science and Engineering, University of Tennessee, Knoxville, TN 37996, USA; 4 Center for Nanophase Materials Sciences Division, Oak Ridge National Laboratory, Oak Ridge, TN 37831, USA; 5 College of Applied Engineering, Sustainability and Technology, Kent State University, Kent, OH 44240, USA; 6 Fibers and textiles Department, University of Georgia, Athens, GA 30602, USA

**Keywords:** carbon nanotube yarns, crosslinking, electron beam, grafting

## Abstract

Carbon nanotube (CNT) yarns exhibit low tensile strength compared to conventional high-performance carbon fibers due to the facile sliding of CNTs past one another. Electron beam (e-beam) irradiation was employed for in a single-step surface modification of CNTs to improve the mechanical properties of this material. To this end, CNT yarns were simultaneously functionalized and crosslinked using acrylic acid (AA) and acrylonitrile (AN) in an e-beam irradiation process. The chemical modification of CNT yarns was confirmed by X-ray photoelectron spectroscopy (XPS), Raman spectroscopy and scanning electron microscopy (SEM). The best improvement in mechanical properties was achieved on a sample treated with an aqueous solution of AA and subsequent irradiation. CNT yarn treatment with AA enhanced the strength (444.5 ± 68.4 MPa) by more than 75% and the modulus (21.5 ± 0.6 GPa) by more than 144% as compared to untreated CNT yarn (strength 251 ± 26.5 MPa and modulus 8.8 ± 1.2 GPa).

## Introduction

Due to their exceptional mechanical, thermal, and electrical properties (Young’s modulus of 1 TPa, tensile strength above 100 GPa), carbon nanotubes (CNTs) are promising materials for various advanced technologies, including CNT-reinforced polymer composites [1–2]. Although many investigations have been carried out with these materials, it still remains a challenge to assemble CNTs in materials on the macroscopic scale [3]. Because of the difficulties in dispersing pristine CNTs in polymers, the assembly of CNTs into macroscopic fibers, with the tubes aligned parallel along the CNT yarn axis, has been focused on [4–13].

There are several methods to grow, align, and fabricate yarns of CNTs [14–16]. Theoretical studies show that CNT yarns can exhibit more than ten times the tensile strength of current carbon fibers [15]. However, CNT yarns prepared commercially in the bulk have only half the tensile strength of conventional high-performance carbon fibers. The reasons for the lower tensile strength compared to carbon fibers are believed to be defects and an inhomogeneous nanostructure, very weak interactions between CNTs, and the packing of CNT bundles [17–18]. It is still a challenge to develop a method that can produce high-strength CNT yarns continuously with industrial scale-up [16]. To date, the solution is to increase the interactions among CNTs in a fiber (covalent bonds among the tubes). In doing so, the sliding of CNTs will be minimized and this may lead to an increase in the mechanical strength of the fiber. Covalent bonding among the tubes can be obtained via simultaneous functionalization and crosslinking [19]. Possible strategies for enhancing the friction between CNTs include modifying CNT surfaces through physical processes (e.g., condensing the tubes under high pressure) [16], chemical processes [20–22], chemical treatment followed by irradiation [23] and radiation processes only [24–26].

Radiation processes (electron beam and gamma irradiation), with the aim of modifying carbon-based materials, have been used for modification of materials and still are the subject of investigations [27–28]. Ionizing radiation has a sufficiently high energy to break bonds and create free radicals that chemically react in several ways over a short period of time. The large penetrating power of high-energy radiation provides an opportunity to carry out grafting at different depths of the substrate and, also crosslinking by introducing junctions between polymer chains and transform a linear polymer into a three dimensional molecule. In general, these junctions may be responsible for the improvement of mechanical properties [29].

In this study, we investigated the morphology and mechanical properties of multiwalled nanotube CNT yarns exposed to electron beam irradiation to, simultaneously, introduce functional groups grafted along the CNT yarn and achieve crosslinking among these functional groups. This strategy is a controlled process that can be integrated easily into a complete process line. This process requires minimal sample preparation, short time of exposure in AA and AN solutions, and it is environmentally friendly.

A preliminary investigation was necessary to verify the multiwall nanotube (MWNT) surface response when mixed with AN solution and irradiated with an e-beam.

## Experimental

### Materials and instruments

CNT yarn (produced through a proprietary process by the University of Cincinnati), acrylic acid (AA, Acros Organics, 98%), acrylonitrile (AN, Acros Organics, 99%), methanol (Fisher Chemicals, 99.8%), tetrahydrofuran (THF, Fisher Chemical, ACS reagent), iron(II) sulfate heptahydrate (FeSO_4_·7H_2_O, Acros Organics, 99+%). All chemical reagents were used as received. The irradiation process was carried out in an industrial accelerator operated by NEO Beam – Mercury Plastics, Inc – Middlefield-OH, 3.8 MeV beam energy, pulse current 38.3 mA, 27 kGy/pass with dose rate of 5 kGy/s.

### Sample preparation

A preliminary investigation was carried out with MWNTs as a loose powder grafted with AN. For this preliminary investigation, MWNTs as a loose powder were exposed to a direct radiation grafting technique in an aqueous solution of AN (20%, v/v in MeOH/H_2_O) and 4% of inhibitor Mohr’s salt ((NH_4_)_2_Fe(SO_4_)_2_·6H_2_O) at a dose of 27 kGy. The low dose of 27 kGy was chosen for this preliminary investigation because only functionalization on the MWNT powder surface was desired. The aim of this preliminary study was to verify the conversion of AN to PAN on the MWNT powder surface under e-beam irradiation. An aqueous solution of AN was placed in a plastic bag, MWNTs were added to it, and the contents were exposed to the electron accelerator to be irradiated. A blank sample was prepared using the same procedure without irradiation. The irradiated MWNTs were washed with copious amounts of THF. The functionalized CNT yarns were vacuum dried at room temperature.

After this preliminary investigation, two sets of CNT yarns were treated with AA and AN, respectively. For the first set of experiment, as-received CNT yarn was immersed in solutions of AA (80% v/v) in MeOH/H_2_O (30% v/v) and the second set of CNT yarn was functionalized with AN (80% v/v) in MeOH/H_2_O (30% v/v). To avoid the homopolymerization process, 4% of inhibitor metal salt (FeSO_4_·7H_2_O) was added to all the solutions.

Immediately after the yarns were immersed in the solution, they were stretched on a cardboard. A light tension was placed on the fibers ends so that the yarn was kept straight during e-beam process. The fibers mounted on cardboard were protected with aluminum foil during the irradiation process. The CNT yarns were irradiated up to 108 kGy and washed with copious quantities of THF. The grafted CNT yarns were vacuum dried at room temperature.

### Characterization

Surface chemical composition and bonding were analyzed by X-ray photoelectron spectroscopy (XPS) using a Thermo Scientific K-Alpha instrument. The K-Alpha uses Al Kα X-rays focused to a spot 400 μm in diameter. Emitted photoelectrons were energy analyzed using a 180° double focusing hemispherical analyzer with a 128-channel detector. Survey data were collected at 200 eV pass energy and an energy resolution of 1 eV/step, while core level data were collected at 50 eV pass energy and 0.1 eV/step energy resolution. Sample charging was eliminated by using the dual-beam charge compensation source of the device, which uses both low energy Ar ions and low-energy electrons. Data were collected and analyzed using the Advantage data system (v.4.61). XPS survey spectra were collected from 0 to 1350 eV.

Raman analyses were carried out in a Horiba Jobin-Yvon T64000 Raman spectrometer equipped with a Peltier-cooled CCD with excellent sensitivity between 200–1000 nm and using a 600 gr/mm grating. The samples were deposited onto a glass slide, and the spectra were collected using a 50× objective in a backscattering configuration. The excitation energy was 2.33 eV from the 532.1 nm line of an argon laser. For each sample, a set of five spectra were collected at different points in the interval from 300 cm^−1^ to 3000 cm^−1^. All spectra were treated to subtract the background and the peaks were fitted using Lorentzian curves.

The tensile properties of CNT yarns were assessed on a MTS single filament tensile tester, with 25 mm gauge length at an extension rate of 0.2 mm/min. Ten specimens from each yarn sample were tested, and the average of the test results is reported. The yarn diameter (*d*) was measured using a 500× optical microscope and was used to calculate the yarn strength (breaking force/0.25π*d*^2^). CNT yarn is cylindrical and the diameter ranges between 50 a 60 µm. The tensile testing of the yarn samples were kept constant in terms of length of 25.4 mm.

The morphology of CNT yarns was investigated using a Zeiss Auriga dual beam focused ion beam (FIB) and scanning electron microscope (SEM) in which electron and ion beam can be used simultaneously. The FIB is generated from a gallium liquid metal ion source with resolution of 7 nm at 30 keV acceleration voltages. The e-beam is generated from field emission gun electron source with high resolution SEM 1 nm at 15 keV and 1.9 nm at 1 keV.

## Results and Discussion

### Preliminary investigation of AN on MWNT by XPS

**Figure 1:**
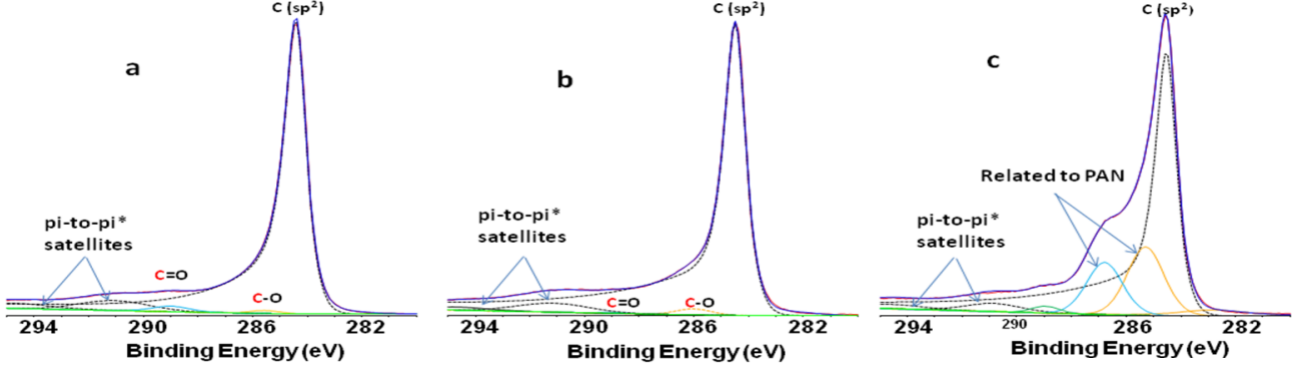
Deconvoluted XPS C 1s spectra of (a) pristine MWNT, (b) MWNT soaked in aqueous solution of 20% AN without irradiation, and (c) MWNT soaked in aqueous solution of 20% AN and irradiated at 27 kGy (c).

The deconvoluted XPS C 1s spectra are shown in Figure 1. The surface composition (atom %) calculated from XPS survey spectra and provided in Table 1 is an important feature to compare non-irradiated and irradiated grafted MWNT powders.

**Table 1:** Surface compositions (atom %) calculated from XPS survey spectra for pristine MWNTs, MWNTs treated with aqueous solution of 20% AN, and MWNTs treated with aqueous solution of 20% AN and irradiated with a dose of 27 kGy.

MWNT sample	C (atom %)	O (atom %)	N (atom %)	Fe (atom %)	Al (atom %)	S (atom %)

pristine	96.3	3.6	0.2	0.0	0.0	0.0
AN 20% – without irradiation	89.5	8.3	0.6	1.0	0.3	0.4
AN 20% – 27 kGy	85.1	8.9	4.5	0.9	0.0	0.6

The significant increase of the N content is an indication of the success of the radiation grafting process in AN as a grafting medium. As is evident from Table 1, both irradiated and non-irradiated MWNTs, showed nitrogen on the surface but the irradiated material showed significantly more (ca. 7.5-times higher content). The O 1s spectra feature a single, broad, asymmetric peak centered at 533.0–533.1 eV related to oxygen group functionality on carbon surfaces. It was observed that there was no significant increase in oxygen content as compared to the blank sample. However, it exhibits greater intensity for the C–O peak (see Figure 1). As also illustrated in Table 1, some traces of contaminant elements (sulfur (S), iron (Fe) and aluminum (Al)) were observed in XPS spectra of all samples, which can be attributed to traces of the inhibitor metal salt, FeSO_4_ and other contaminants. The samples exhibit a shake-up satellite peak (π→π*, 291.3 eV) characteristic of aromatic C structures. The peak with binding energy (BE) of 284.1 eV corresponds to non-functionalized sp^2^ carbon atoms in the CNT structure. In addition, the BE position and width of the peaks were similar and consistent with those of polyacrylonitrile (PAN) from the literature (Figure 1c).

This process relies on the fundamentals of radiation grafting polymerization. The advantage of the process is that an initiator is not required, avoiding the formation of free radicals on the substrate backbone/monomer, contamination and problems with local heating of the initiator. Basically, the e-beam is absorbed by all elements of the system, while water usually absorbs most of this energy [30–32]. The irradiation of water produces hydrogen atoms, solvated electrons, hydroxyl radicals, H_2_, H_2_O_2_, H_3_O^+^ and OH^−^ [33]. The hydroxyl radicals can be immobilized on the MWNT surface and this produces trapped radicals on the surface of the MWNTs. As a result, the trapped radicals on the MWNTs surface act as initiators for graft polymerization of AN on the MWNT surface. On the other hand, the unsaturated C=C from vinyl monomers degrades easily under the radiation process. An excess of inhibitor may lead to diffusion through the yarn and the Fe^2+^ ions may deactivate the free radicals trapped on the MWNT surface.

At this point, it should be noted that, to date, there are some previous reports on the use of radiation grafting polymerization to functionalize graphitic nanostructures [30,33–34]. This kind of grafting remains the subject of investigation because of a variety of structural transformations that may occur in carbon nanostructures under irradiation and different experimental configurations producing interesting and unexpected results. Evora et al. investigated the functionalization of vapor-grown carbon fibers (VGCFs), via gamma and e-beam radiation processes using acrylic acid as a source of oxygen functional groups, which enhanced the dispersion of VGCFs in water [33]. Unlike CNTs, VGCFs have a unique morphology, a tubular structure with the sidewalls composed of angled graphite sheets. In addition, VGCFs are different from CNTs in the method of production and they have few functional groups at the edges [35].

### CNT yarns functionalized with AN and AA irradiated at 108 kGy

**Figure 2:**
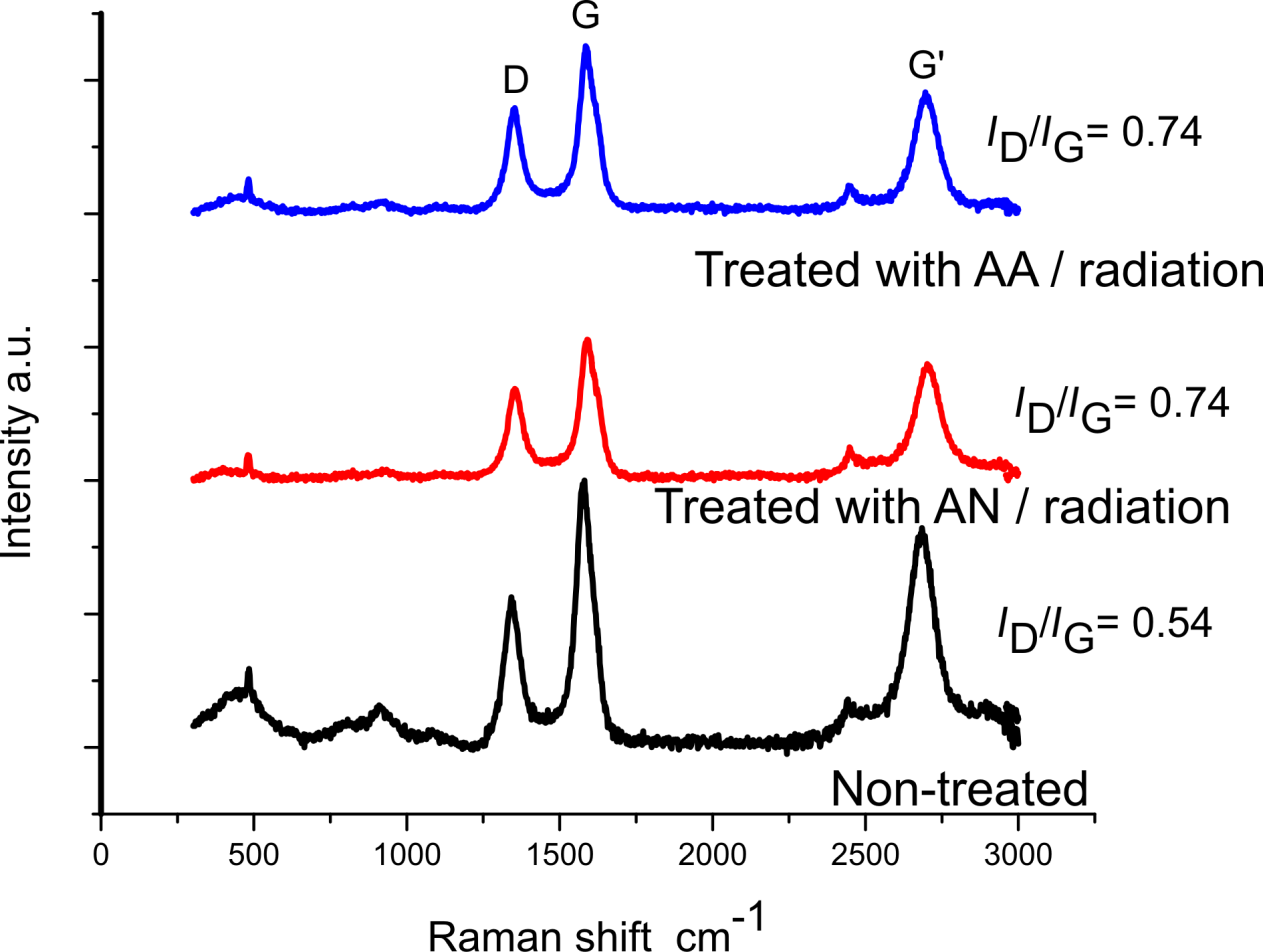
Raman spectroscopy results for CNT yarn treated with aqueous solutions of 80% AN and 80% AA and irradiated at 108 kGy.

Raman spectroscopy is a useful method to investigate the covalent sidewall modification in nanotube functionalization chemistry. The results for untreated CNT yarn and samples treated with monomers and irradiated are shown in Figure 2. The Raman spectra were normalized on the basis of the G-band intensity. For carbon materials, Raman spectra contain two intense bands between 1000 cm^−1^ and 2000 cm^−1^. The peaks around 1580 cm^−1^ and 1350 cm^−1^ correspond to the G band (C=C in-plane stretching mode) and D band (disorder), respectively. The D band originates from hybridized vibrational mode associated with graphene edges. It indicates the presence of some disorder in the graphene structure. The degree of disorder in sp^2^-hybridized carbon materials is given by the intensity ratio between the D band and the *G* band (*I*_D_/*I*_G_) [36]. This ratio is thus useful to investigate the functionalization process.

The broad band around 500 cm^−1^ observed in the spectra of the untreated samples may be due to the presence of amorphous carbon. Carbon atoms can be sputtered from the outer shell under irradiation and recombine. Fitting of all spectra was achieved with Lorentzian peaks and the *I*_D_/*I*_G_ ratio was calculated for each sample. *I*_D_/*I*_G_ increases with an increase in the number of defects on the sidewall. Chemical functionalization introduces sp^3^-defects and disrupts the π–π conjugation of the graphitic structure. As shown in Figure 2, this functionalization process occurred in the CNT yarn structure. The *I*_D_/*I*_G_ ratio increased from 0.54 for untreated CNT yarn to 0.74 for the CNT yarns treated with AA and AN.

**Figure 3:**
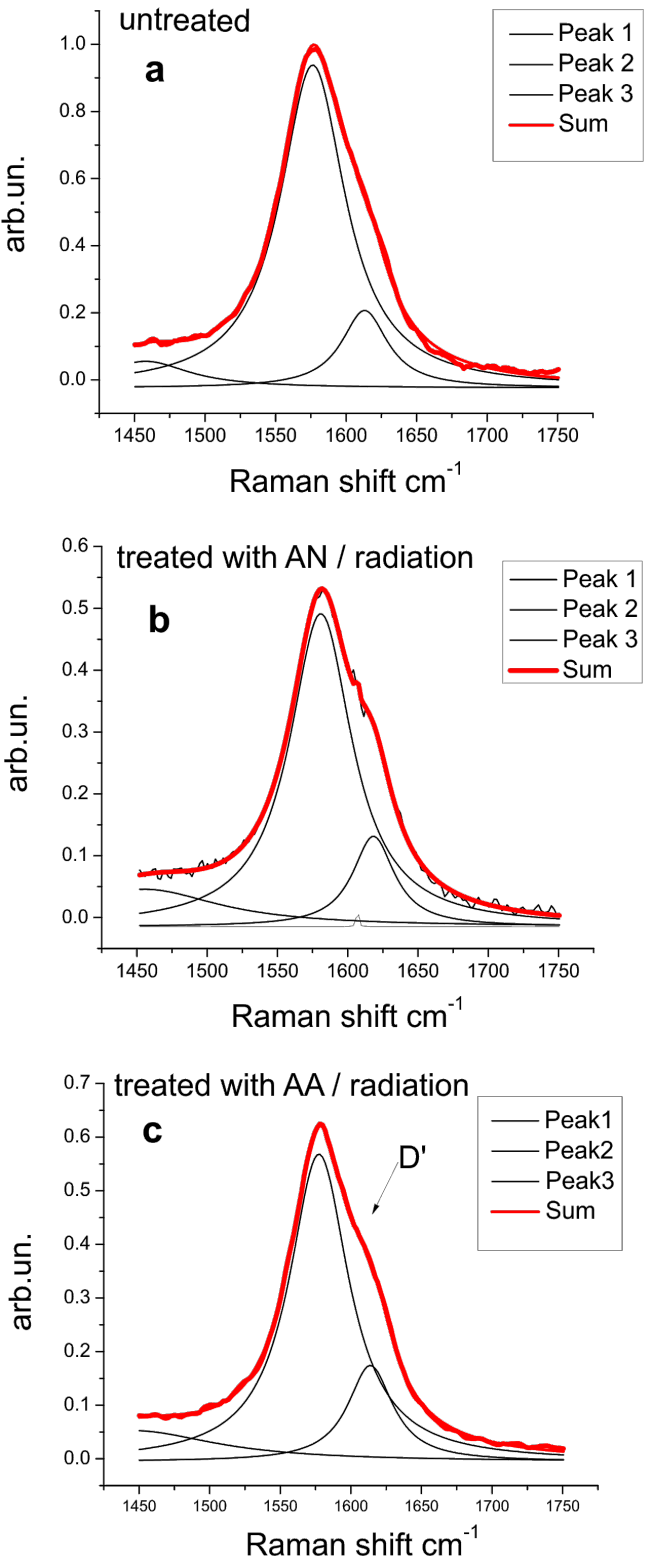
G′ peaks of (a) untreated CNT yarn, (b) CNT yarn treated with AN and radiation, and (c) CNT yarn treated with AA and radiation.

Another peak that has a significant intensity in graphene structures is the G′ band. The G′ band is a single peak in single-layer graphene, whereas it splits into four peaks in bilayer graphene, reflecting the evolution of the electron band structure [37–38]. However, the G′ band is not only present in monolayer graphene, but also in carbon structures that have turbostratic graphite (TGr) as part of their components, such as MWNTs. This can be confirmed by the Raman spectra presented in Figure 2. Both structures, monolayer and TGr, are represented as Lorentzian peaks, however, TGr has a larger line width. The reason for this similarity is that there is no interlayer interaction between the graphene planes in TGr. The full width at half maximum (FWHM) of the G′ peak gives information about the level of graphitization of the material because the G′ band is correlated to the electronic band structure of the graphitic material. There was a decrease of the G′ peak FWHM from 117.51 cm^−1^ (untreated sample) to 109.51 cm^−1^ (treated with 80% AN and irradiated with a dose of 108 kGy) and 105 cm^−1^ (treated with 80% AA and irradiated with a dose of 108 kGy) (Figure 3). This happens because some of the graphitic structure was damaged due to covalent bond formation with functional groups originating from the vinyl monomers.

Additionally, to support this conclusion, a shoulder known as the D′ peak was observed around 1613 cm^−1^. For the treated samples, this peak became more prominent than that of the untreated samples (Figure 3). Shi et al. also observed this in their investigation of grafting poly(styrene-*co*-acrylonitrile) onto MWCNTs. The presence of the shoulder is an indication of an increase in the number of defects in the graphite structure [39]. These results are in agreement with the *I*_D_/*I*_G_ ratio and it is probably related to the breakage of the C=C bonds in the graphitic wall through e-beam irradiation. The growth of polymer chains is initiated at these sites [40].

**Figure 4:**
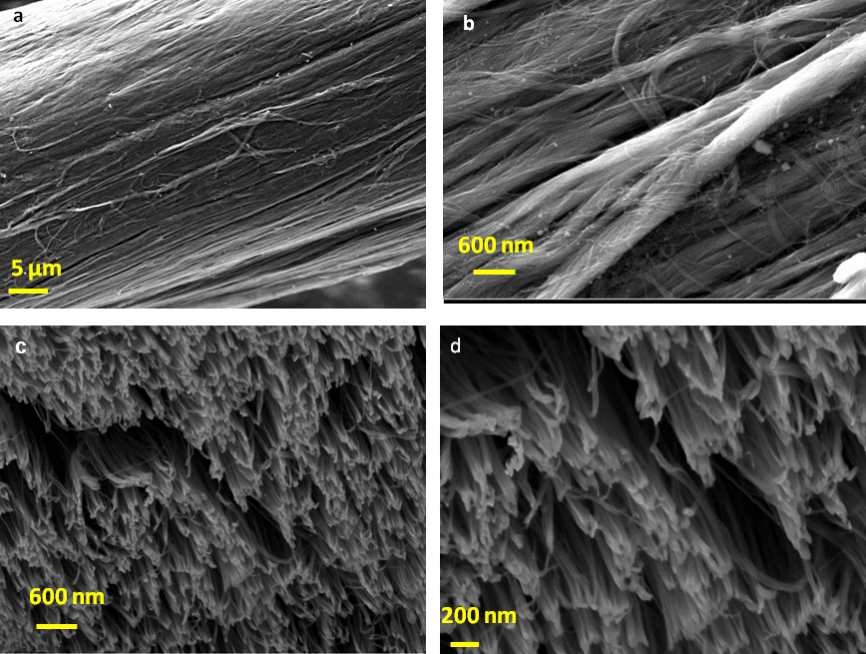
(a,b) SEM images of untreated CNT yarn; (c,d) FIB images at different magnifications of untreated CNT yarn.

**Figure 5:**
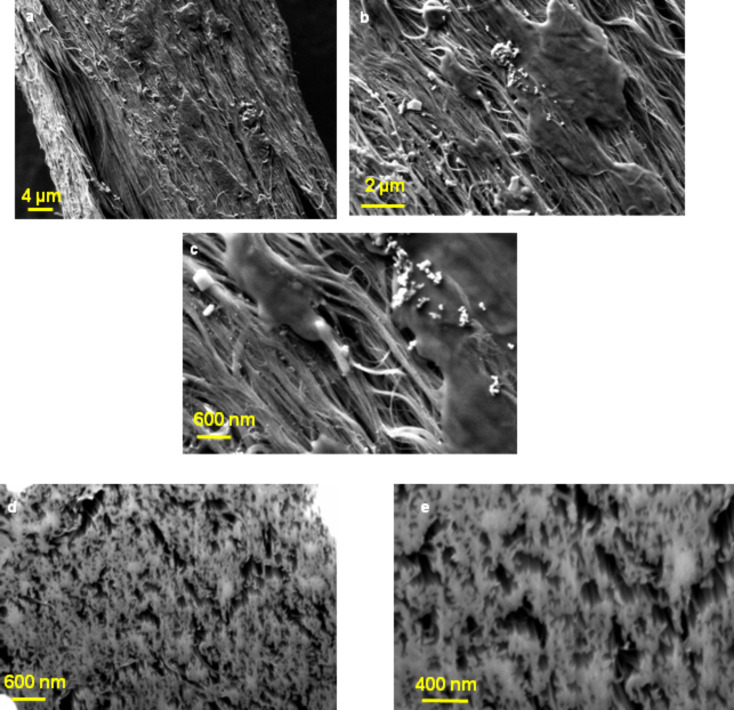
(a–c) SEM images of CNT yarn treated with 80% PAN (same area and at different magnifications). (d,e) FIB images at different magnifications of a CNT treated with PAN.

**Figure 6:**
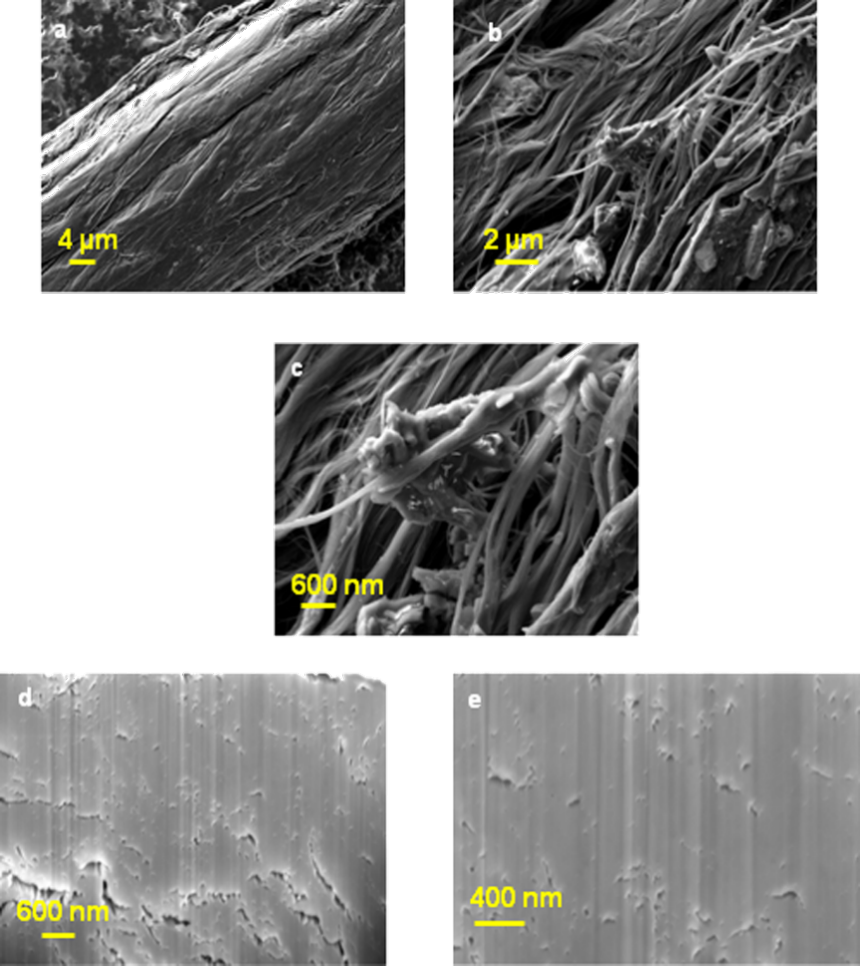
(a–c) SEM images of CNT yarn treated with 80% AA (same area at different magnifications). (d,e) FIB images at different magnifications of a CNT treated with AA.

Figure 4, Figure 5 and Figure 6 show SEM images of untreated CNT yarn and CNT yarns treated with monomers and the e-beam process. The comparison of the SEM images gives a clear indication of the change in the morphology of the modified samples. Both SEM images of treated CNT yarns showed a considerable deposition of PAN (see Figure 5a–c) and polyacrylic acid (PAA) on their surface (see Figure 6a–c). The CNT yarn treated with AN and irradiation showed a rougher surface. The CNT yarn treated with AA exhibited a smoother appearance but wavy extensions protrude from the surface as shown in Figure 6. This is probably related to hydrophobic interactions, van der Waals and capillary force interactions between the MWCNTs and the repulsion of functional groups [41]. On the FIB images, it is evident that the simultaneous process proposed in this report occurs inside the fiber structure (Figure 5d,e and Figure 6d,e). Figure 4c,d show FIB images of untreated samples and show a CNT yarn with many open spaces between the aligned MWCNTs. The main force that keeps them together is van der Waals forces, which is a weak force. On the other hand, the FIB image of the PAN sample (Figure 5d,e) shows a higher density than untreated yarn (Figure 4c,d), which may be attributed to simultaneous grafting and crosslinking taking place. Therefore, the modification happens not only on the surface but also inside the structure of the fiber. The same phenomenon is observed in the FIB images of the samples modified with AA, as presented in Figure 6d,e. Although the FIB image of the yarn modified with AA shows some cracks, it is noticed that there is a complete interaction between the CNT yarn surface and the PAA leading to a good adhesion. There are almost no empty spaces between MWCNTs and PAA. The modification happens on the surface as well as inside of the fiber.

In this study, CNT yarns treated with AA and AN and irradiated with an e-beam exhibit improved mechanical properties because of the crosslinking process. The mechanism of crosslinking along with the grafting process occurs through the formation of free radicals between the polymer chains grafted on the CNT walls. The radiolysis of water molecules present in the system plays an important role in the crosslinking process. The hydroxyl radicals originating from the water attack the polymer chain grafted on the CNT yarn walls, resulting in the formation of radicals, which will form crosslinks between the polymer molecules [42].

Polymer infiltration methods have been reported to enhance the strength as well, but they can be time-consuming. Jung et al. studied the effect of polymers on the structure of the CNT yarns. The CNT yarn was soaked in polymer solution to achieve full solvent and polymer infiltration. The CNT yarn was then dried in a vacuum oven at 100 °C for more than 6 h. They found some improvement in mechanical properties of CNT yarn infiltrated with polymers [43]. Hiremath et al. also concluded the same when they investigated the effect of toluene and polystyrene infiltrated in the microstructure of carbon nanotube yarns [44].

**Figure 7:**
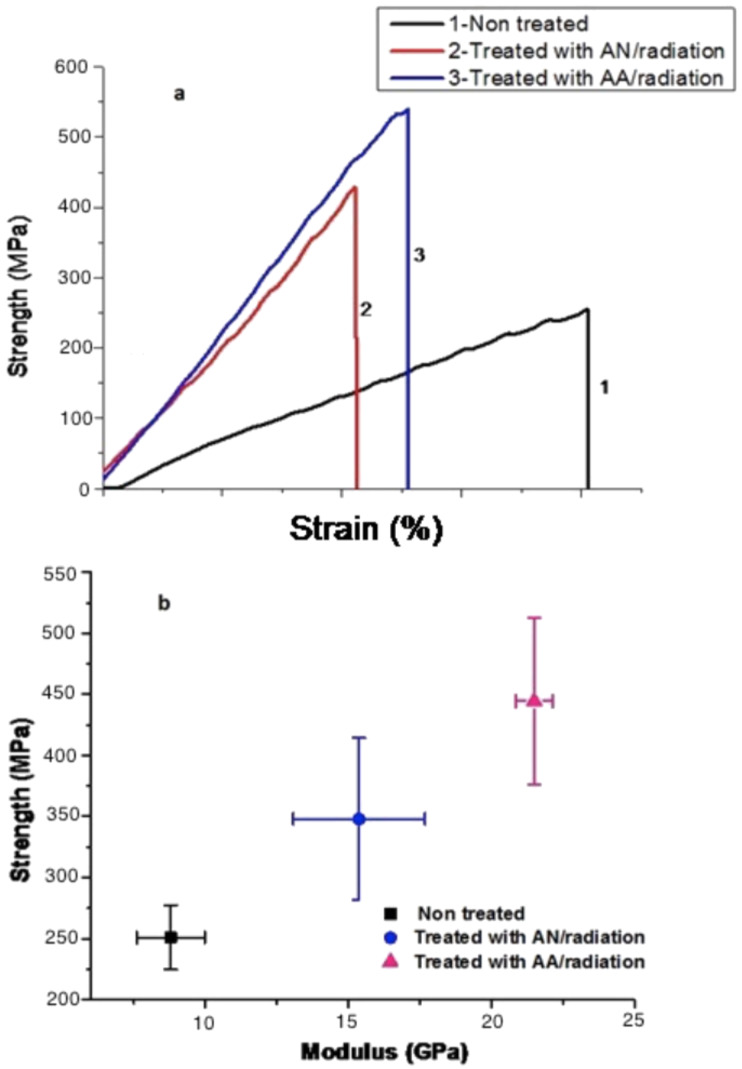
(a) Typical stress–strain curves of untreated CNT yarn and CNT yarn treated with AN and AA and radiation; (b) tensile strength versus modulus of untreated CNT yarn and CNT yarn treated with AN and AA and radiation.

The results of strength and modulus measurements are presented in Figure 7. The typical stress–strain curves for non-functionalized CNT yarn and CNT yarn functionalized with AN and AA are given in Figure 7a. The slopes of the curves increase for CNT yarns functionalized and crosslinked, which implies that the elastic modulus increases with crosslinking while the strain decreases significantly when CNT yarns are modified. Untreated samples exhibit lower strength but higher strain because of slippage of the CNTs. The strain is reduced due to enhancement in the CNTs interaction because of the crosslinking process. After the treatment of the yarn samples, load transfer and strength increase.

The crosslinking process strongly affects the strength and elastic modulus [45]. The untreated CNT yarn demonstrates a strength of 251.09 ± 26.52 MPa and a modulus of 8.79 ± 1.19 GPa The modification of the CNT yarn structure with PAN improved the strength to 347.94 ± 66.36 MPa and the modulus to 15.38 ± 1.19 GPa, because of the crosslinking that probably occurred at the nitrogen atom of the =C=N– functionality [46]. These results show an increase of about 38.6% in strength and about 75% in elastic modulus.

The biggest improvement in mechanical properties was achieved with the process using an aqueous solution of AA at a concentration of 80%, which agrees with FIB images presented in Figure 6d,e. The increase in packing between MWCNTs caused the improvement of strength and modulus. The resulting samples treated with AA showed an increase of the strength to 444.51 ± 68.45 MPa, which represents an increase of ca. 77.03%, and of the modulus to 21.5 ± 0.65 GPa, which represents an increase of ca. 144.60% when compared to the untreated samples. The improved mechanical properties are attributed to formation of crosslinks among the polymer grafted onto the surface. The PAA can form crosslinks by anhydride formation [42]. In an acid solution, the recombination process becomes very fast and crosslinking dominates over scission. These radicals, induced by radiolysis on the PAA structure can recombine and form a 3D crosslinked network [47].

## Conclusion

CNT yarns exhibit low tensile strength compared to conventional high-performance carbon fibers due to the facile sliding of CNTs past one another. We used the vinyl monomers, AA and AN, to modify CNT yarn via a single-step process using an electron beam as radiation source. This process simultaneously introduced PAN and PAA along the CNT yarn and caused crosslinking among these functional groups, leading to the improvement of mechanical properties. The Raman and SEM results showed that the polymer was covalently bonded and crosslinked onto the CNT yarn structure. The value of *I*_D_/*I*_G_ increased showing an increase of defects on the sidewall due to chemical functionalization, which introduced sp^3^-defects and disrupted the π–π conjugation of the graphitic structure. The FIB images showed that the radiation grafting polymerization and crosslinking process took place over all the structure of the CNT yarns. The best improvement in mechanical properties was achieved using an aqueous solution of AA at a concentration of 80%. The CNT yarn modified with PAA and crosslinked showed a complete interaction between the CNTs and the PAA, which can explain their considerable improvement in mechanical properties. Overall, the treated fibers exhibit improved mechanical properties in comparison with the untreated fiber. The samples treated with AA exhibit an increase in strength from 251.09 ± 26.52 MPa for the untreated CNT yarn to 444.51 ± 68.45 MPa and modulus from 8.79 ± 1.19 GPa to 21.5 ± 0.65 GPa.
